# Cell Versus Cytokine – Directed Therapies for Hemophagocytic Lymphohistiocytosis (HLH) in Inborn Errors of Immunity

**DOI:** 10.3389/fimmu.2020.00808

**Published:** 2020-05-08

**Authors:** Oliver Wegehaupt, Katharina Wustrau, Kai Lehmberg, Stephan Ehl

**Affiliations:** ^1^Center for Chronic Immunodeficiency, Medical Center, Faculty of Medicine, Institute for Immunodeficiency, University of Freiburg, Freiburg, Germany; ^2^Center for Pediatrics, Faculty of Medicine, Medical Center – University of Freiburg, University of Freiburg, Freiburg, Germany; ^3^Division of Pediatric Stem Cell Transplantation and Immunology, University Medical Center Hamburg-Eppendorf, Hamburg, Germany

**Keywords:** hemophagocytic lymphohistiocytosis, inborn errors of immunity, pathogenesis, therapy, cytokine, inflammation, HSCT

## Abstract

Hemophagocytic lymphohistiocytosis (HLH) is a heterogeneous hyperinflammatory syndrome with different pathways of pathogenesis resulting in similar clinical presentations. It is best defined and understood if presenting in the context of genetic immunodeficiencies associated with defects of lymphocyte cytotoxicity. In these “primary” forms of HLH, cellular and soluble immune effectors are relatively well characterized. While etoposide-based broad cell-directed therapies remain standard of care, more specific therapies targeting these effectors individually are increasingly available. Anti-CD52 as a cell-directed therapy and anti-IFN-gamma, IL-18BP, and JAK-inhibition as cytokine-directed therapies are expected to broaden the therapeutic options, but the precise role of these drugs in first-line and rescue treatment indications remains to be defined. A number of additional inborn errors of immunity are associated with episodes of immune activation fulfilling the clinical criteria of HLH. Impaired pathogen control is a key driver of hyperinflammation in some conditions, while others are characterized by a strong autoinflammatory component. This heterogeneity of disease-driving factors and the variable severity in disease progression in these conditions do not allow a simple adaptation of protocols established for “primary” HLH to HLH in the context of other inborn errors of immunity. Cytokine-directed therapies hold significant promise in these increasingly recognized disorders.

## Primary Hemophagocytic Lymphohistiocytosis

**FIGURE 1:**
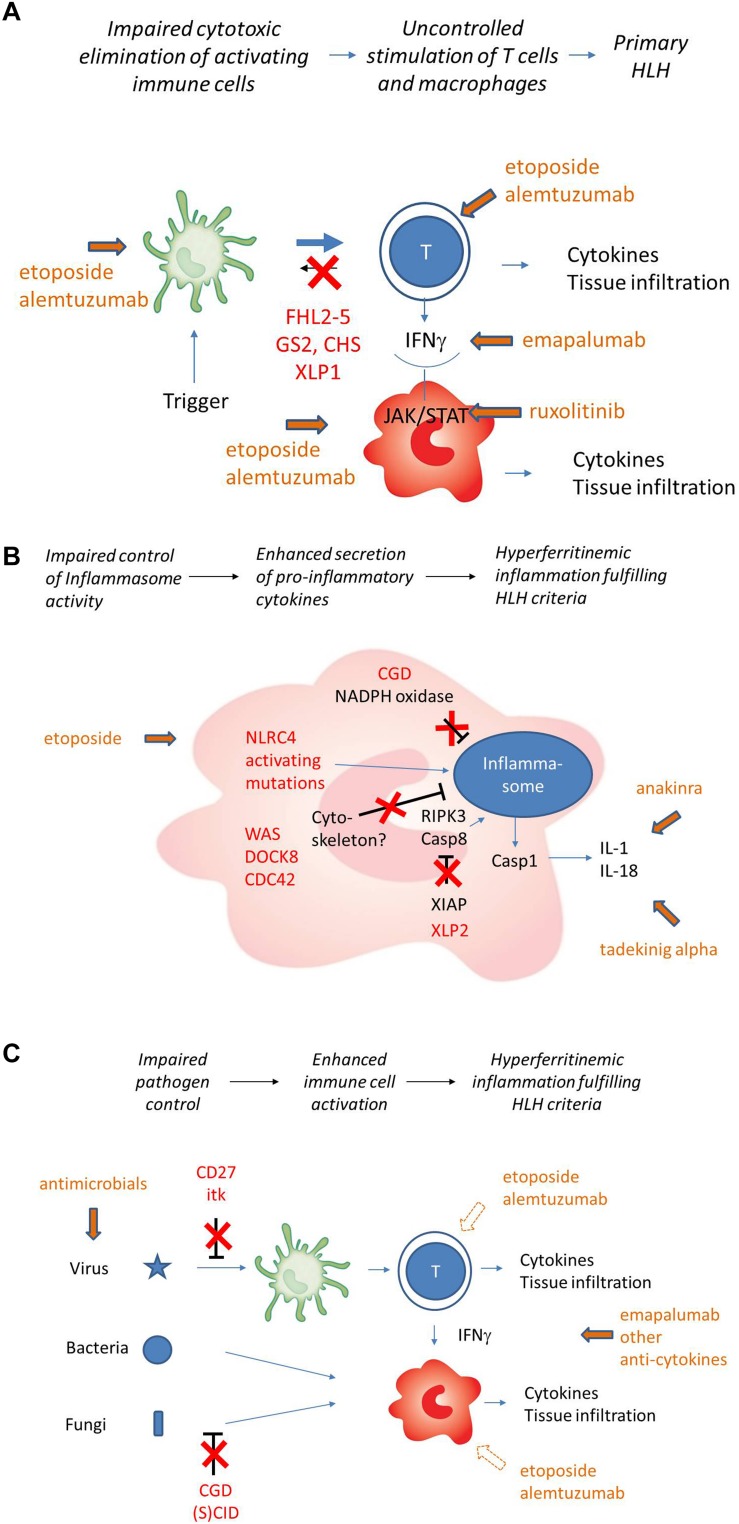
**(A)** Pathogenesis of “primary” HLH (simplified). Impaired cytotoxicity (red) leads to uncontrolled T cell activation by APC. T cell secreted IFNg is the key driver of macrophage activation. Cellular and cytokine targets of therapy are indicated in green. **(B)** Pathogenesis of HLH in the context of impaired inflammasome homeostasis (simplified). Inborn errors of immunity shown (NLRC4, XIAP) or assumed (Cytoskeletal disorders, CGD) to be involved in inflammasome homeostasis are indicated in red. Cytokine targets of therapy are indicated in green. Macrophages are the key cells involved, T cells play a less prominent role. Cell-directed therapies are rarely used. **(C)** HLH pathogenesis in the context of inborn errors of immunity with impaired pathogen control (simplified). Inborn errors of immunity impairing virus control (mainly EBV) and/or bacterial/fungal control are indicated in red. Subsequent immune stimulation leads to hypersecretion of variable cytokines. Macrophage activation can occur in the absence of T cells, but T cells can be involved depending on the genetic defect and the trigger. Cell-directed therapies further impair pathogen control and should only be used in exceptional cases.

Hemophagocytic lymphohistiocytosis (HLH) is a highly inflammatory syndrome with uncontrolled, excessive immune activation. HLH is the key manifestation in a range of autosomal-recessive genetic diseases defined as familiar forms of HLH (FHL). FHL includes FHL1 to FHL5 (OMIM #267700, #603553, #608898, #603552, and #613101) caused by defects in lymphocyte cytotoxicity affecting perforin or proteins involved in the exocytosis of perforin-containing lytic granules (degranulation deficiencies) (Table 1). It was first described in 1952 as familial hemophagocytic reticulosis (1). In FHL2 patients with “null” mutations, the first manifestation of disease symptoms is in most cases observed in the first 6 months of life, but may already be present *in utero* or at birth (2). HLH tends to occur later in patients with other FHL variants (3, 4) and in patients with biallelic “hypomorphic” mutations and an initial HLH episode has been reported as late as 63 years of age (5). The incidence of FHL is estimated at 1:50,000–1:100,000 (6, 7). Some genetic immunodeficiency diseases associated with pigment dilution such as Griscelli syndrome type II (GS-II; OMIM # 607624) and Chediak-Higashi syndrome (CHS; OMIM #214500) are also caused by degranulation defects (8). The similar pathogenesis and the frequent occurrence of HLH in these conditions allow their classification as “primary” HLH (Figure 1A).

**TABLE 1:** Genetically determined forms of hemophagocytotic lymphohistiocytosis (HLH).

**Primary HLH**	**Gene**	**Protein**	**Pathophysiology**	**Functional testing**
**Familial HLH (FHL)**				
FHL-1	Unknown	Unknown		
FHL-2	PFR1	Perforin	Lack of perforin expression in lytic granules	Perforin expression
FHL-3	UNC13D	Munc13-4	Deficiency in fusion of lytic granule with plasma membrane	Degranulation
FHL-4	STX11	Syntaxin11	Deficiency in fusion of lytic granule with plasma membrane	Degranulation
FHL-5	STXBP2	Munc18-2	Deficiency in fusion of lytic granule with plasma membrane	Degranulation
**Other immunodeficiency syndromes with defect in degranulation**				
GS-II	RAB27A	Rab27a	Deficiency in docking of lytic granule to the plasma membrane	Degranulation hair microscopy
CHS	LYST	Lyst	Defect in maturation of vesicles into secretory cytotoxic granules	Degranulation hair microscopy
—				

**Other inborn errors of immunity**	**Gene**	**Protein**	**Pathophysiology**	**Functional testing**

**Immunodeficiency syndromes with HLH as a frequent manifestation**				
XLP-1	SH2D1A	SAP	Defective killing of EBV infected B-cells by CD8 and NK cells	SAP expression
XLP-2	BIRC4	XIAP	Impaired inhibition of inflammasome activity	XIAP expression L18MDP assay
TIM3 deficiency	HAVCR2	TIM3	Persistent T cell activation and increased production of inflammatory cytokines	TIM3 expression
**Immunodeficiency syndromes with HLH as an occasional manifestation**				
Chronic granulomatous disease (CGD)	CYBB, CYBA, NCF1, NCF2, NCF4	Components of NADPH oxidase	Excessive inflammatory responses due to altered inflammasome regulation by NADPH oxidase?	Oxidative Burst
(S)CID	>50 genes	various	Lack of pathogen control	Lymphocyte phenotyping
Wiskott-Aldrich syndrome	WAS	WASP	Lack of pathogen control Impaired cytoskeleton-inflammasome interaction?	WASP expression (FACS)
CD27 deficiency	CD27	CD27	Impaired co-stimulation of T cells Lack of EBV control	CD27 expression
ITK deficiency	ITK	ITK	Impaired TCR mediated signaling Lack of EBV control	ITK expression
IFNγ receptor deficiency	IFNGR1 IFNGR2	IFN-gamma receptor	Lack of pathogen control (mycobacteria, salmonella)	STAT1 phosphorylation
ALPS	FAS (het) FASLG	FAS FASLG	Defects in Fas ligand-mediated elimination of activated lymphocytes	TCR DNT Vitamin B12, soluble FasL
**Autoinflammatory diseases with HLH as a frequent manifestation**				
NLRC4 gain of function	NLRC4 (het)	NLRC4	Constitutive inflammasome activation IL-1β/IL-18 production	Genetic testing
CDC42 mutations	CDC42 (het)	CDC42	Impaired cytoskeleton-inflammasome interaction?	Genetic testing

## Pathophysiological Basis of “Primary” Hemophagocytic Lymphohistiocytosis

FHL2-5, GS-II, and CHS all affect the cytotoxic granule-mediated cell death pathway (8, 9). Under physiological conditions, immune stimulation such as a viral infection leads to priming of cytotoxic T-lymphocytes by APC, followed by their activation and proliferation. These activated T cells and NK cells can recognize virus-infected target cells and subsequently eliminate these through polarized release of perforin- and granzyme-containing granules (10, 11). Entry of granzymes into target-cells by membrane-pores established by perforin activity mediates apoptotic cell death. Notably, this cytotoxic activity is also directed against APC, providing an important negative feedback-loop that limits T cell activation (12).

In “primary” HLH, deficient cytotoxic activity of CTL and NK cells impairs the timely elimination of APCs. Their persistence leads to continuous T-cell stimulation. Incessantly activated T-cells infiltrate tissues and release various pro-inflammatory mediators, in particular interferon-gamma, a potent macrophage-stimulating cytokine (13). Continuous macrophage activation, in turn, further fuels release of a broad range of inflammatory cytokines such as IL-1, IL-6, IL-18, and TNF-alpha (14–16) and leads to tissue infiltration of macrophages and hemophagocytosis. Since in the course of an immune response, T cells themselves can also become targets of the cytotoxic activity of NK cells and T cells, lack of this control mechanism may further impair immune homeostasis (17, 18). Clinical manifestations of HLH are mainly a result of tissue infiltration by T cells and macrophages and the accompanying excessive cytokine storm.

This model of “primary” HLH pathophysiology has mainly been established in key studies in cytotoxicity deficient mice that develop all clinical features used for the diagnosis of HLH in patients upon persistent wide-spread systemic infection with lymphocytic choriomeningitis virus (19). In most patients with “primary” HLH, no persistent systemic viral infection can be demonstrated (2), asking for a note of caution whether this model really explains all aspects of the human disease.

## Other Inborn Errors of Immunity Predisposing to HLH: Pathogenetic Heterogeneity

In a group of additional inborn errors of immunity, HLH occurs less frequently, although it can still be the presenting clinical manifestation. In these diseases, HLH pathogenesis is variable and mostly different from that of “primary” disease (Table 1). A brief review of current understanding of pathogenesis of these diseases is relevant for the discussion of therapeutic approaches.

Two X-linked genetic diseases predispose to HLH predominantly in the context of EBV infection (20) (Figure 1B). XLP1 (OMIM #308240) is caused by defects in SAP, a small adaptor protein that regulates signaling in T and NK cells by binding to the SLAM family of signaling receptors (21). Many aspects of XLP1 pathogenesis can be explained by impaired T/NK-B cell interaction. As a consequence, affected patients frequently suffer from hypogammaglobulinemia and its infectious consequences due to impaired T cell help to B cells and lymphomas due to impaired control of malignant B cells (22). Cerebral vasculitis and aplastic anemia can also be life-threatening manifestations. Poor T/NK-cell mediated control of EBV-infected B cells, in part linked to impaired activation of 2B4 (a SLAM receptor) mediated cytotoxic function, is the basis of HLH, that develops in about 30% of XLP1 patients (23).

XLP2 (OMIM #300079) is caused by defects in XIAP, a protein with antiapoptotic functions, regulatory functions for autophagy and control functions for inflammasome activity (24, 25). It also modulates the NOD1/NOD2 pathways which contribute to intracellular sensing of bacterial infection. A link to lymphocyte cytotoxicity has not been established. Important clinical manifestations of XLP2 are early onset inflammatory bowel disease, splenomegaly and periodic fever (26). The pathogenesis of mostly EBV-induced HLH, which occurs in more than 30% of patients, is unclear. However, an autoinflammatory component due to dysregulated NLRP3 inflammasome activation is reflected by excessive levels of free serum IL-18 and is more prominent than in “primary” HLH (27). Notably, some, but not all biological activities of IL-18 are mediated by IFNγ (28). The frequency of HLH in XLP1 and XLP2 has led to their inclusion in the classification of “primary” HLH and the therapeutic principles of FHL have also been successfully used to treat HLH in XLP (20, 29). However, both XLP variants have a pathophysiology that is clearly different from “primary” HLH and this may offer different treatment options. This is particularly relevant for treatment of manifestations different from HLH in these conditions.

TIM3 deficiency (OMIM #618398) caused by *HAVCR2* mutations is another autosomal-recessive inborn error of immunity that predisposes to HLH in a particular context, i.e., in subcutaneous panniculitis T cell lymphoma (SPTCL) (30). TIM3 is an inhibitory molecule expressed mainly on T cells and NK cells, but also on myeloid cells. TIM3 mutations causing aberrant protein folding and lack of surface expression lead to an autoinflammatory and autoimmune phenotype with hyperactivated myeloid cells producing high levels of IL-1 and IL-18 and uncontrolled CD8 T cell proliferation (31). This promotes SPTCL formation and its association with HLH.

Heterozygous NLRC4 gain-of-function mutations (OMIM #606831) lead to constitutive activation of the NLRC4 inflammasome resulting in enterocolitis and macrophage activation associated with a clinical picture of HLH. It is characterized by excessive levels of free IL-18 and IL-1beta (32, 33). Heterozygous mutations in CDC42 affecting amino acids 186, 188, or 192 also lead to a hyperinflammatory syndrome including neonatal cytopenias, hepatosplenomegaly, recurrent febrile episodes and urticaria-like rashes that can fulfill HLH criteria. This autoinflammatory disease is also characterized by very high levels of IL-18 and IL-1beta, suggesting dysregulated inflammasome function (34). The mutations are postulated to interfere with actin assembly, thus affecting signaling, cytoskeletal rearrangement and cell migration. All three conditions are characterized by a significant autoinflammatory disease component that calls for treatment approaches different from primary HLH (Figure 1B).

Finally, immune activation fulfilling the clinical criteria of HLH occasionally occurs in several additional primary immunodeficiencies, including SCID, some combined immunodeficiencies such as Wiskott-Aldrich syndrome, CD27 deficiency and ITK deficiency, chronic granulomatous disease (CGD) and IFNγ receptor deficiency (35, 36) (Figures 1B,C). The examples of SCID and IFNγR deficiency illustrate that the clinical syndrome of HLH as defined by the HLH-2004 clinical criteria requires neither T cells nor IFNγ, illustrating that this form of HLH is different from “primary” HLH. In fact, the HLH-like immune activation in these diseases is in most cases due to impaired pathogen control and rather represents an infection-induced HLH. Additional factors such as altered inflammasome regulation by NADPH oxidase in CGD (37) and potentially impaired cytoskeleton – inflammasome cross-talk in patients with WAS, DOCK8 deficiency and CDC42 mutations likely also contribute (38–40). Overall, these examples illustrate that also in familial HLH cases, a careful characterization of the genetic disorder underlying HLH is required as it allows to choose treatment targeted at the specific pathogenesis.

## Therapeutic Strategies

The heterogeneity in pathophysiology of “primary” HLH caused by cytotoxicity defects versus HLH associated with other inborn errors of immunity makes it obvious that there is no “one fits all” therapeutic strategy. Treatment must be targeted to the pathophysiology and results from treatment studies obtained in one group of diseases cannot simply be transferred to another. Therapeutic regimens in primary HLH are either directed at the immune cells involved, i.e., APC, T cells and macrophages, or at the cytokines secreted by these cells. The goal is to disrupt ongoing immune stimulation and to limit severe hyperinflammation and tissue damage. The implementation of broad cell-directed therapies was critical to improve survival in this life-threatening condition (41). However, more specific anti-cellular therapies and therapeutic targeting of particular key cytokines and their downstream effects are currently evaluated in clinical trials. In the absence of published data on several of these novel approaches, this review can only point out the therapeutic principles and indicate which trials to watch as they have the potential to impact on standard-of-care within the next 5 years.

Overall, the therapeutic approach to primary HLH can be divided into four main phases:

(1) Induction of remission.
(2) Control of triggers.
(3) Maintenance of remission and salvage therapy.
(4) Curing the underlying condition.

## Induction of Remission

Timely treatment of HLH is essential for prognosis. Untreated patients with active “primary” HLH show a survival of approximately 2 months due to progressive organ failure (42). Delayed initiation of therapy increases the risk of neurological complications. In most cases, initial decisions must be made in the absence of a confirmed genetic diagnosis, but tests of protein expression and degranulation are rapidly available and have high sensitivity and specificity for “primary” HLH (43–45). Important differential diagnosis requiring different treatment approaches such as malignancy or metabolic disease should be considered (46, 47). Leishmaniosis must be ruled out in all patients with a plausible risk (48).

## Targeting Cells

For decades, first-line therapy for primary HLH has been centered on cell-oriented approaches. The widely used standard-of-care is based on the dexamethasone/etoposide-based HLH-94 and HLH-2004 studies. A consensus statement addressing various aspects of its use in detail has recently been published by the HLH Steering Committee of the Histiocyte Society (49).

### Etoposide-Based Protocols

**FIGURE 2:**
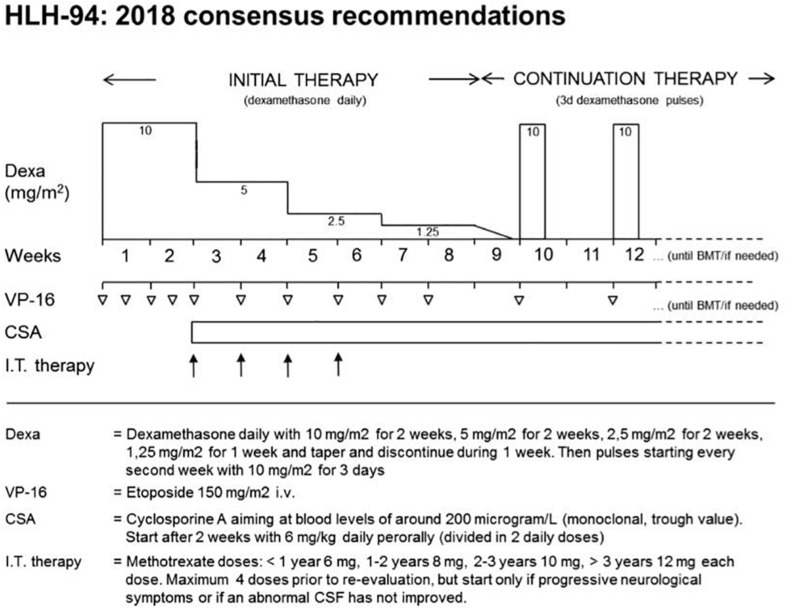
2018 consensus statements by the HLH Steering Committee of the Histiocyte Society recommending the use of HLH-94. The HLH-94 protocol is based on immunochemotherapy including dexamethasone, etoposide, and cyclosporine A (CSA). After an intensive phase of 2 weeks with high doses of dexamethasone and twice weekly administration of etoposide, dexamethasone is tapered until week nine. Cyclosporine A is used from week three onward to prevent reactivation. Intrathecal therapy with methotrexate is recommended in patients with CNS involvement. Immunological testing and genetic confirmation of the underlying genetic disease is required in all patients and should provide the basis for HSCT within 8 weeks. Copyright Clearance Center’s RightsLink^®^ service/Elsevier.

The HLH-94 protocol is based on immuno-chemotherapy including dexamethasone, etoposide and CSA to achieve remission of the hyperinflammatory state and to maintain remission until HSCT can be performed (41). Functionally, all agents target lymphocytes, macrophages and antigen presenting cells. The cytostatic agent etoposide induces cell death mainly in activated T cells (50), but also in macrophages and dendritic cells. The use of the calcineurin inhibitor CSA leads to an inhibition of the transcription factor NFAT (nuclear factor of activated T-cells) and thus to a reduced activation and proliferation of T cells. Steroids slow down inflammation by reducing cytokine secretion, but in addition, they have a moderate cytotoxic effect on activated T cells. It is recommended to treat patients with CNS involvement also with intrathecal methotrexate (51), although there is no clear evidence of benefit. The protocol has a 2-week intensive phase with dexamethasone and twice weekly administration of etoposide, followed by 6 weeks of weekly etoposide and steroid tapering. In this second phase, CSA is used to prevent reactivation (49). Rapid immunological testing followed by genetic confirmation of the underlying genetic disease is required in all patients and should provide the basis for HSCT within these 8 weeks (Figure 2). In the international multicenter registry-based HLH-2004 study, 5-year probability of survival for children with genetically verified familial HLH treated with this protocol was 59% (52). Dexamethasone/etoposide-based protocols have been successfully used in XLP or patients with TIM3 deficiency. It remains an ultimate choice also in other inborn errors of immunity, but the toxicity and immunosuppression associated with etoposide asks for more targeted therapies in these conditions.

### Antithymocyte Globulin (ATG)

Antithymocyte globulin directly targets T cells and other lymphocytes, to a minor extent also granulocytes and monocytes (53). In a retrospective single-center analysis of 38 patients with familial hemophagocytosis, a protocol consisting of steroids, CSA and first-line ATG resulted in a higher initial remission rate compared to the HLH-94 protocol (active disease in 26% of patients versus 53%, after 2 months of therapy) but was associated with a higher percentage of relapses before HSCT (32% versus 13%) (54). In an attempt to combine advantages of both protocols, a trial combining ATG and etoposide has been performed (55), but the results have not yet been reported. There is no clear role for ATG beyond “primary” HLH.

### Alemtuzumab (Anti-CD52)

More recently, the humanized monoclonal anti-CD52 antibody alemtuzumab has been used in patients with “primary” HLH (56). It is directed against the CD52 antigen (CAMPATH 1) which is a surface protein on mature lymphocytes and APCs. First promising results have been achieved when used in a bridging to transplant setting (57). Excellent initial results have been orally reported from a trial evaluating Alemtuzumab as first-line treatment for “primary” HLH (in combination with methyl-prednisolone and CSA) (58). The profound and long-lasting immune suppression and limits this drug to “primary” HLH, where induction of remission is rapidly followed by HSCT. The problem of viral (re-)activation is an important caveat when using alemtuzumab in XLP.

## Targeting Cytokines

In a disease associated with excessive production of a large number of cytokines, it is not self-evident that blockade of a single cytokine should have significant therapeutic effects. However, pivotal studies in mouse models of “primary” HLH have indicated that some key cytokines, in particular IFN-gamma, are drivers of the immune dysregulation (60) and that their neutralization can interrupt the inflammatory circle and restore immune homeostasis (52, 59). As a consequence, therapeutic approaches targeting IFN-gamma, its induction and its downstream effects have emerged as promising strategies that are at different stages of evaluation in clinical trials of primary HLH.

### Interferon-Gamma

Emapalumab is a recombinant human monoclonal antibody against interferon gamma (60). It has received FDA approval in November 2018 for the treatment of pediatric and adult patients with primary HLH with refractory, recurrent, or progressive disease or intolerance to HLH therapy (see section salvage therapy). An international multicenter follow-up study to further assess the efficacy and safety of emapalumab is still ongoing (61). This trial will also provide data on its use as first-line therapy in “primary” HLH. Serum levels of CXCL9 emerge as an interesting biomarker for increased IFNγ activity (62) and may be particularly helpful when considering the use of emapalumab in first-line treatment of HLH in the context of other inborn errors of immunity. Notably, a patient with CDC42 mutation who did not respond to steroids, CSA, and anakinra was successfully treated with emapalumab (34).

### JAK-Inhibition

Janus kinase inhibitors represent interesting therapeutic compounds in the context of HLH, since they not only inhibit signaling downstream of IFN gamma, but also of several other pro-inflammatory cytokines. In the mouse-model of LCMV-induced “primary” HLH, the disease manifestations, including CNS involvement, were reduced upon JAK1/2 blockade by ruxolitinib (63, 64). The successful individual use of ruxolitinib reported in single cases of secondary HLH (65–67) has resulted in its prospective evaluation for this indication (68). Preliminary results of a single-center phase 2 pilot study on the efficacy of ruxolitinib in secondary HLH demonstrate good tolerance to ruxolitinib in a small cohort of five patients (69). However, “primary” HLH is excluded in these studies. A trial investigating the benefit of JAK inhibition in first-line treatment of human “primary” HLH is in preparation. We are not aware of reports on the use of JAK inhibitors in HLH in the context of other inborn errors of immunity, but this is a plausible pathway to explore.

### Targeting IL-18

IL-18 is released by activated macrophages and can induce IFN-gamma and other pro- inflammatory cytokines (28). Several reports have found elevated free IL-18 concentrations (i.e., IL-18 not bound to its binding protein IL-18BP) in the serum of patients with both “primary” and secondary HLH as well as in animal models and IL- 18 levels correlated with the presence of HLH-criteria and disease progression (70). In a murine model of “primary” HLH it was shown that treatment with IL-18BP can reduce severe organ damage, but does not improve survival (71). An ongoing multicenter, double-blind, placebo-controlled, randomized withdrawal trial evaluates efficacy and safety of IL-18BP (tadekinig alfa) in pediatric patients with NLRC4 associated hyperinflammation including HLH or XIAP deficiency, diseases, in which IL-18 levels are particularly elevated (73). While this treatment seems promising to attenuate the autoinflammatory manifestations of XIAP deficiency, it remains to be seen whether it also has a role in acute EBV-induced HLH in this disease.

### IL-1, IL-6, and TNF Alpha Blockade

The pro-inflammatory cytokines elevated in “primary” HLH also include IL-1, IL-6, and TNF alpha, which can be targeted by monoclonal antibodies and other blocking agents. They have been used successfully in the context of secondary HLH (72, 74) and related conditions associated with a “cytokine storm” such as hyperinflammation associated with CAR-T cell therapy (75). Moreover, case reports have illustrated a partial effect of IL-1 blockade in patients with NLRC4 or CDC42 mutations (32, 34) and in patients with CGD (76). Anecdotal reports have reported efficacy of IL-6 blockade in manifestations of XIAP deficiency different from acute EBV-induced HLH (77). However, efficacy of IL-1, IL-6, or TNF alpha blockade in “primary” HLH has not been clearly documented, neither in first-line nor in rescue therapy.

## Control of Triggers

Infections should be diagnosed and treated aggressively in all forms of HLH. When active EBV infection is present, rituximab (anti-CD20 antibody) can help controlling the immune stimulation by eliminating EBV infected B cells (78, 79). However, in some states of persistent EBV replication, EBV has been demonstrated in T or NK cells leading to resistance against rituximab treatment (80). Cell-targeted therapy results in significant immunosuppression, such that reverse isolation, aspergillus-effective antifungal and PCJ prophylaxis should be administered (81). Weekly monitoring for infection or reactivation of latent pathogens (EBV, CMV, adenovirus, aspergillus antigen) is recommended (49).

## Monitoring Treatment Response

Monitoring response to therapy and detecting early signs of reactivation is crucial in patients with “primary” HLH (79, 82). The response of cytopenia is a sensitive parameter to judge treatment response (83). Since neutropenia frequently occurs treatment-related, thrombocytopenia is the more valuable parameter. Bone marrow puncture can be of some help in distinguishing between the activity of HLH and the myelotoxic side effect of therapy. Ferritin usually shows a significant decrease in the first days of successful treatment. However, complete normalization of ferritin can take weeks and can be even further delayed by the transfusion of erythrocytes (84). sCD25 is more dynamic, but it may still take a few days until a substantial decrease can be observed. In patients with initially low fibrinogen, this parameter can be used together with transaminases and coagulation studies to assess the treatment response (83). Other biomarkers for disease activity such as free IL-18 or CXCL9 are being explored. If more rapid turnaround times can be achieved, they might be valuable for guiding therapy in the future.

## Salvage Therapy in Refractory HLH

Early mortality of acute HLH remains a major concern. 25–50% of patients with acute “primary” HLH fail to achieve rapid and sustained initial remission after etoposide-based therapy. If cytopenia [in particular thrombocytopenia <40 G (G/L)] and ferritin and/or sCD25 fail to respond after 2 weeks, the risk for an adverse outcome increases, justifying consideration of alternative (salvage) therapy (49). There are no standard recommendations for the treatment of relapsing or refractory HLH. The salvage therapies published so far include alemtuzumab, anakinra, ATG, and regimens consisting of liposomal doxorubicin, etoposide, and dexamethasone (85). In an observational study reporting on treatment of 22 patients with refractory HLH with alemtuzumab, 86 percent of patients showed partial response, and 77 percent were able to receive HSCT (86). Notably, CMV and adenovirus viremia occurred in 23–32% of patients.

Emapalumab, a neutralizing antibody against INFγ, has recently been licensed as the first drug for the treatment patients with “primary” hemophagocytic lymphohistiocytosis (HLH) with refractory, recurrent or progressive disease or intolerance with conventional HLH therapy (87). The recommended starting dose is 1 mg/kg twice per week with dexamethasone as a background treatment, but doses can be increased up to 10 mg/kg based on clinical response (88). Due to the risk of serious infections (frequent during therapy of primary HLH patients and observed in 32% of patients in the trial) patients should receive prophylaxis for Herpes Zoster, Pneumocystis jirovecii, and fungal infections and should be monitored for tuberculosis, adenovirus, EBV and CMV.

The study included 27 patients with a mean age of 1 year (range: 0.1 to 13 years), with a “primary” HLH in 82% of patients. Patients had received various combinations of dexamethasone, etoposide, CSA, and anti-thymocyte globulin prior to emapalumab. Full response was defined as normalization of all, while partial response was defined as normalization of ≥3 HLH parameters and HLH improvement was defined as ≥3 HLH abnormalities improved by at least 50% from baseline. Twenty patients completed the 8-week study, while seven were prematurely withdrawn. Seventy percent (19/27) of patients proceeded to HSCT. The overall response rate was 63%, the median time to response was 8 days. A complete response was achieved in 7 patients, partial response in 8 patients and HLH improvement in 2 patients (88, 89). Since refractory primary HLH has a dismal prognosis, these data are encouraging. However, the exact place of this drug in the context of existing and emerging therapies remains to be defined.

## Definitive Therapy

### Hematopoietic Stem Cell Transplantation (HSCT)

To prevent recurrences, allogenic stem cell transplantation should be carried out as soon as possible after achieving initial remission in the “primary” HLH (49, 90). It remains the only curative option. The timing of HSCT has to balance the risks between achieving full remission versus reactivation. Although active disease at conditioning remains a risk factor, full remission of all clinical symptoms is not required for successful HSCT. In particular, active neurological disease should prompt aggressive management including early HSCT (51).

Allogeneic HSCT is also the definitive treatment of choice for HLH in the context of several other inborn errors of immunity, including XLP1 or XLP2 and patients with TIM3 deficiency (91, 92). Furthermore, HSCT has been successfully performed in a patient with a CDC42 mutation (34). However, there has been no report of HSCT in NLRC4 deficiency, where it is unlikely to impact on IL-18 hypersecretion by intestinal epithelial cells (93). Careful and broad genetic and functional evaluation is therefore mandatory before proceeding to HSCT based on clinical grounds in rare cases of familial or recurrent HLH without detection of a genetic cause.

In cases with suspected “primary” HLH, donor search and pretransplantation diagnostics should be carried out promptly during the initial presentation. Bi-allelic mutations should be ruled out in potential related donors. In autosomal-recessive disease, heterozygous carriers are in most cases appropriate donors. In the X-linked conditions, skewed X-inactivation should be excluded in potential female carrier donors (94). Conditioning regimes for HSCT in “primary” HLH have been discussed elsewhere.

### Gene Therapy

For genetic diseases manifesting in hematopoietic cells, hematopoietic stem cell gene therapy is an important option (95). Preclinical murine studies in a perforin knock-out mouse showed a correction of the HLH phenotype after lentiviral gene therapy of autologous hematopoietic stem cells (96). However, high levels of expression were necessary to fully correct the HLH phenotype (97). Successful gene transfer into hematopoietic stem cells has also been demonstrated in a mouse model (98) and in patient T cells with MUNC13-4 deficiency (99). Furthermore, correction of cellular and humoral immune function was achieved by gene therapy in the murine model of XLP1 (100). Since these mice do not develop HLH, the question of whether the gene therapy can fully control the risk of HLH could not be addressed. These preclinical proof-of-concept studies show the therapeutic potential of gene therapy in “primary” HLH and it will be important to see them further investigated in clinical trials in the future.

## Special Situations in “Primary” HLH

### CNS Involvement and Isolated CNS-HLH

Central nervous system involvement is a common complication in “primary” HLH (30–73%) and leads to increased morbidity in long-term survivors (51, 101). Irritability, seizures, meningisms, focal deficits, or reduced level of consciousness are observed in active HLH. Diagnostic parameters of CNS involvement include variable combinations of elevated protein or cell count (>5 cells/μl), lymphocytic pleocytosis, activated monocytes and hemophagocytosis in the CSF. MRI brain morphology can demonstrate cerebral atrophy, diffuse white matter irregularities and multiple focal lesions (102–104). Delayed start of treatment for “primary” HLH increases the risk of neurological complications and is associated with worse CNS outcomes (105).

“Primary” HLH can also present as isolated CNS disease in the absence of any systemic manifestations. These occur particularly in older patients with hypomorphic mutations (106–110), most commonly in patients with FHL2 or Griscelli syndrome (111). Isolated CNS disease has also been documented in patients post-transplant with partial donor chimerism (112, 113). Systemic HLH-directed therapies can improve CNS-HLH unless irreversible damage has already occurred (51). Considering its value in other inflammatory brain diseases, alemtuzumab may provide an interesting option. In the further course, patients with isolated CNS-HLH are at risk for developing full-blown systemic HLH. Allogeneic HSCT is therefore also recommended in patients with isolated CNS disease (111).

### Pre-emptive HSCT

Unless transplanted, all patients with “primary” HLH have a risk of developing life-threatening HLH at any time throughout their life. This risk has to be weighed against the risk of HSCT on an individual basis. In any case, genetic testing of family members, particularly of siblings should be offered promptly after diagnosis of the index case. In a recent analysis of 64 children with primary HLH (index cases), 32 asymptomatic carriers were identified. 16 of 22 asymptomatic carriers received pre-emptive transplantation, of which 15 are alive and in complete remission after 39 months of median follow-up. Eight-year probability of survival was significantly higher than that in index cases and survival in asymptomatic carriers receiving HSCT before disease activation was significantly higher than in those receiving HSCT after HLH activation (93% versus 64%) (114). Hence, most experts recommend pre-emptive HSCT for FHL unless mutations are very mild.

## Outlook

In the last few decades, significant progress has been made in understanding the genetic basis and pathogenesis of HLH in the context of inborn errors of immunity. This has set the stage for rapid diagnosis and a more targeted therapy of this serious clinical condition. The outcome of “primary” HLH has significantly improved with cell-targeted therapies. New cytokine-directed treatments will increase the therapeutic flexibility, but it remains to be seen whether they will show enough efficacy to fully replace this aggressive approach. In the emerging field of HLH associated with other inborn errors of immunity, established and novel cytokine-directed therapies are expected to become the treatment of choice.

## Author Contributions

OW wrote the manuscript. KW, KL, and SE contributed to manuscript revision and approved the submitted version.

## Conflict of Interest

SE is part of Advisory Boards at SOBI, UCB, and Novartis and has received research support from UCB. KL is part of an Advisory Board at SOBI.

The remaining authors declare that the research was conducted in the absence of any commercial or financial relationships that could be construed as a potential conflict of interest.

## Abbreviations

APC: antigen-presenting cell
ATG: antithymocyte globulin
CMV: cytomegalovirus
CNS: central nervous system
CSA: cyclosporine A
EBV: Epstein-Barr virus
FHL: familial hemophagocytotic lymphohistiocytosis
HLH: hemophagocytotic lymphohistiocytosis
HSCT: hematopoietic stem cell transplantation
JAK: janus kinase
MRI: magnetic resonance imaging
NK: natural killer cell
RIC: reduced-intensity-conditioning
VOD: veno-occlusive disease
XLP: X-linked lymphoproliferative syndrome.
